# Heterologous prime-boost cellular vaccination induces potent antitumor immunity against triple negative breast cancer

**DOI:** 10.3389/fimmu.2023.1098344

**Published:** 2023-02-13

**Authors:** Seyedeh-Raheleh Niavarani, Guillaume St-Cyr, Lauren Daniel, Christine Lawson, Hugo Giguère, Almohanad A. Alkayyal, Lee-Hwa Tai

**Affiliations:** ^1^ Department of Immunology and Cell Biology, Université de Sherbrooke, Sherbrooke, QC, Canada; ^2^ Department of Medical Laboratory Technology, Faculty of Applied Medical Sciences, University of Tabuk, Tabuk, Saudi Arabia; ^3^ Immunology Research Program, King Abdullah International Medical Research Center, Riyadh, Saudi Arabia; ^4^ Centre de Recherche du Centre Hospitalier de l’Université de Sherbrooke, Sherbrooke, QC, Canada

**Keywords:** Immunogenic cancer vaccine, oncolytic virotherapy, triple negative breast cancer, tumor microenvironment, immune effector cells

## Abstract

**Introduction:**

Triple negative breast cancer (TNBC) is the most aggressive and hard-to-treat subtype of breast cancer, affecting 10-20% of all women diagnosed with breast cancer. Surgery, chemotherapy and hormone/Her2 targeted therapies are the cornerstones of treatment for breast cancer, but women with TNBC do not benefit from these treatments. Although the prognosis is dismal, immunotherapies hold significant promise in TNBC, even in wide spread disease because TNBC is infiltrated with more immune cells. This preclinical study is proposing to optimize an oncolytic virus-infected cell vaccine (ICV) based on a prime-boost vaccination strategy to address this unmet clinical need.

**Methods:**

We used various classes of immunomodulators to improve the immunogenicity of whole tumor cells in the prime vaccine, followed by their infection with oncolytic Vesicular Stomatitis Virus (VSVd51) to deliver the boost vaccine. For in vivo studies, we compared the efficacy of a homologous prime-boost vaccination regimen to a heterologous strategy by treating 4T1 tumor bearing BALB/c mice and further by conducting re-challenge studies to evaluate immune memory responses in surviving mice. Due to the aggressive nature of 4T1 tumor spread (akin to stage IV TNBC in human patients), we also compared early surgical resection of primary tumors versus later surgical resection combined with vaccination.

**Results:**

*In vitro* results demonstrated that immunogenic cell death (ICD) markers and pro-inflammatory cytokines were released at the highest levels following treatment of mouse 4T1 TNBC cells with oxaliplatin chemotherapy and influenza vaccine. These ICD inducers also contributed towards higher dendritic cell recruitment and activation. With the top ICD inducers at hand, we observed that treatment of TNBC-bearing mice with the influenza virus-modified prime vaccine followed by VSVd51 infected boost vaccine resulted in the best survival. Furthermore, higher frequencies of both effector and central memory T cells along with a complete absence of recurrent tumors were observed in re-challenged mice. Importantly, early surgical resection combined with prime-boost vaccination led to improved overall survival in mice.

**Conclusion:**

Taken together, this novel cancer vaccination strategy following early surgical resection could be a promising therapeutic avenue for TNBC patients.

## 1 Introduction

The incidence of triple negative breast cancer (TNBC) is significant, with approximately 230,000 cases diagnosed globally each year (World Health Organization 2020). TNBC is a heterogeneous group of breast cancers that do not express estrogen, progesterone and HER-2 receptors. Compared to other breast cancer subtypes, TNBC has a poor prognosis due to a high rate of early recurrence, distant metastasis and lack of targeted therapies (1). Neoadjuvant chemotherapy and surgical resection are recommended for early stage TNBC. A pathologic complete response to this frontline care is the best indicator of long-term survival (2, 3). For patients with late stage, metastatic or chemo-resistant TNBC, there are no further, effective treatment options.

The presence of tumor infiltrating lymphocytes (TILs) within tumor tissue, suggests an immune response to tumor-associated antigens (TAA). The presence of high levels of TILs in TNBC tissue and its correlation with improved prognosis has been recently evaluated (4–6). Moreover, both genomic instability and high rates of genetic mutations have been found in TNBC tissue, which implicates the production of neoantigens and increased tumor immunogenicity (7, 8). While TNBC contains more pre-existing TILs than other breast cancer subtypes, these TILs reveal dysfunction (5, 9–12). Possible reasons include deficient Interferon (IFN) signaling and immune suppressive pathways in the tumor microenvironment (TME) that dampen TIL responses (13, 14).

Oncolytic viruses (OV) are a form of immunotherapy that uses attenuated viruses to selectively replicate in and destroy cancer cells, with subsequent induction of anti-tumor immune responses within the TME (15). Many recent studies demonstrate the ability of OV to induce immunogenic cell death (ICD) upon tumor killing (14, 16–18). Infection of tumors by OV leads to the release of pathogen associated molecular patterns (PAMPs) and danger associated molecular patterns (DAMPs), which signal through toll-like receptors (TLR) and activate cellular stress. This results in the production of type I IFN, upregulation of surface major histocompatibility complex (MHC), expression of costimulatory ligands CD80/CD86, and “eat-me” signals such as externalized calreticulin (CRT), high mobility group box 1 (HMGB1), ATP and heat-shock proteins, which are all associated with ICD (19). This leads to the release of TAAs in a T cell inflamed TME capable of recruiting and activating antigen presenting cells (APCs), such as dendritic cells (DC) and anti-tumor CD8^+^ T cells (14, 18, 20, 21).

The lack of precision therapies for TNBC and the existence of pre-existing, but dysfunctional TIL, provides a strong rationale to treat TNBC with immunotherapies, such as therapeutic cancer vaccines. The field of therapeutic cancer vaccines has experienced a revival in research activity in the past decade due to a better understanding of the TME, the breadth of TAA and the development of novel platforms for TAA delivery. Currently, the use of cancer vaccines against TAA such as MAGE-A, NY-ESO-1, and MUC-1 are being tested in preclinical and early stage human trials to activate TNBC TILs (22). Recent gene expression data reveal elevated expression of these TAA in TNBC compared to other breast cancer subtypes (6, 9). Given that TNBC is a highly heterogeneous disease grouped together due to an absence of tissue histological markers, it is reasonable to postulate that not all TNBC patients will response to a cancer vaccine formulation that targets a single TAA. In contrast, treatment with autologous (self) tumor cells will uncover their own complete and individualized tumor antigen repertoire. Significantly, the personalized cancer vaccine may contain many self-tumor antigens that can activate a broad and polyclonal immune response that is able to recognize and remove a distinct population of heterogeneous TNBC tumors (23).

To overcome the immune suppressive TME and induce strong immune responses against TAA and/or neoantigens, we developed an infected cell vaccine (ICV) platform for TNBC. This consists of harvesting tumor cells, followed by their *ex vivo* infection with a replicating OV and direct intratumoral delivery into the TME. This method circumvents the main obstacles to systemic delivery of OV and permits additional *ex vivo* alteration of the tumor cells to enhance immune recognition (17, 24, 25). Autologous tumor cell vaccines are an antigen agnostic form of personalized immunotherapy and uncovers a TNBC patient to their complete and personalized TAA repertoire, therefore lessoning the chances of heterogenous tumor immune evasion (23). Clinical studies on autologous tumor cell vaccines have reliably demonstrated that patient survival is significantly improved when they produce an immune response against their own tumor cells (26, 27). The solid immunological basis supporting cytokine-based tumor cell vaccines propels the development of novel vaccines. Recently, Gradalis and Vaccinogen Inc. are conducting Phase III clinical studies founded on promising clinical responses in breast and colon cancers (26, 28).

We have previously demonstrated that oncolytic rhabdovirus-based ICV can effectively treat preclinical models of colon cancers that are completely resistant to systemic OV treatment, even when the same rhabdovirus is used (24). In a preclinical model of TNBC, we have recently showed that the *in vitro* infection of 4T1 cells with oncolytic vesicular stomatitis virus (VSVd51, a rhabdovirus) results in features of ICD, including enhanced exposure of CRT and release of HMGB1 and ATP. From *in vivo* experiments, we observed enhanced DC, natural killer (NK) and CD8^+^ T cell recruitment and activation, both systemically and in spontaneous metastases to the lungs in ICV treated cohorts. Importantly, we detected improved survival in the presence of an intact immune system in ICV treated mice (17).

To further focus the immune response on TNBC TAA/neoantigens, we are developing a heterologous prime-boost strategy in which flu vaccine infected irradiated whole tumor cells (prime vaccination) followed by the ICV (boost vaccination) is administered to enhance the exposure of the immune system to the same TAA/neoantigen epitopes, but minimizing the anti-OV response. This strategy is different from heterologous prime-boost vaccines used to elicit broadly neutralizing antibodies against infectious diseases (22, 23), where several strains of an inactive virus (with different epitopes) are used to boost the immune response (24). In this study, we used various classes of immunomodulators to improve the immunogenicity of whole tumor cells in the prime vaccine, followed by their infection with VSVd51 to deliver the boost vaccine. We compared the efficacies of homologous prime-boost vaccination versus the heterologous prime-boost strategy by treating 4T1-tumor bearing BALB/c mice and further by conducting an *in vivo* tumor challenge study to assess tumor memory responses. Furthermore, we confirmed that survival can be additionally improved when the prime-boost vaccination strategy is combined with early surgical resection of primary tumor.

## 2 Methods

### 2.1 Cell lines, viruses and ICD inducers

4T1 and MDA-MB-231 cell lines were cultured in DMEM; and BT-549 in RPMI, all supplemented with 10% heat inactivated (HI) FBS + 100U/ml penicillin and 100μg/ml streptomycin (P/S). Cell lines were purchased from ATCC in the past year. All cells were tested for mycoplasma infection, had negative test results and reveal appropriate microscopic morphology at time of experimentation. VSVd51 expressing GFP reporter protein was grown on Vero cells and purified using Opti-Prep purification methods. Viral titers were obtained by a standard plaque assay as previously published (24). Viral cytotoxicity was assessed on the indicated cell lines, and cell viability was carried out as described previously (24). The following ICD inducers were used at their IC50 doses: Oxaliplatin (30 μg/ml, Sigma), Doxorubicin hydrochloride (25 nM, Sigma), BCG vaccine (1X, OncotTice - Merck Inc), seasonal flu vaccine (1X, Sequirus), Td Adsorbed (1X Sanofi Pasteur).

### 2.2 Mice

Female BALB/c mice (6-8 weeks old, 20-25g) were purchased from Charles Rivers (Quebec). Animals were housed in pathogen-free conditions at the Pavilion for Applied Cancer Research of the Université de Sherbrooke with liberal access to food and water. Animals were euthanized by cervical dislocation under anesthesia. All studies were performed in accordance with Université de Sherbrooke guidelines and the Canadian Council on Animal Care. The protocol 2020-2606 was approved by the Faculty of Medicine and Health Sciences Animal Care Committee.

### 2.3 4T1 syngeneic mouse model with resection and prime-boost vaccination

We have previously established a mouse model of spontaneous and aggressive metastasis and surgical resection of Stage IV TNBC (29, 30). 1x10^5^ 4T1 cells in 100μl of sterile 1X PBS at >98% viability were implanted orthotopically into the 4th mammary fat pad of BALB/c mice at day 0. Subsequently, mice were monitored daily by finger palpation of the primary tumor site, the volume of tumors was measured by a digital Vernier caliper and the tumor volume was calculated using the equation (width^2^*length)/2. A complete resection of the primary tumor was performed (tumor volume = 75-80mm^3^) at days 8-10. During surgery, mice were kept under anesthesia (3% induction, 1.5% maintenance of isoflurane with 2% O_2_). For perioperative pain management, mice were injected with 0.05 mg/kg of buprenorphine 1h before and 4h following surgery. Mice were randomized into different cohorts for treatment. At 1-2d post-surgery, mice received 1 dose of irradiated 4T1 cells treated with the best ICD inducers (FLU or Oxa) as the prime vaccine, injected subcutaneously into the cleared surgical bed. One week later, mice in the prime-boost group or the boost control group were treated with irradiated 4T1 cells infected with 10 MOI of VSVd51. Surviving mice were re-challenged by inoculating 1x10^5^ 4T1 cells in the opposite mammary fat pad. The size of re-challenged tumors was measured by digital caliper and the tumor volumes were calculated as described above.

### 2.4 Prime boost vaccine preparation

Prime vaccines were prepared using ICD inducers described above at their IC50 doses. 5x10^6^ of viably dissociated, single cell suspensions of 4T1 primary tumors were γ-irradiated at 50Gy. These parameters has been previously determined to create a non-proliferating, but intact whole cell vaccine (25). Cells were then treated with ICD inducers for 24h at 37°C, harvested and washed twice prior to resuspension in 100μl and injected into the cleared mammary fat pad. ICV using VSVd51 was prepared as previously published (24). VSVd51 was added to the cells at 5x10^7^ PFU and further incubated at 37°C for 24h. This preparation was injected in the cleared mammary fat pad in mice at 100μl, giving each mouse 1:10 γ-irradiated cells to virus per dose (10 multiplicity of infection, MOI). Peripheral immune cells were assessed 1 week following boost vaccination.

### 2.5 Flow cytometry

To measure mouse spleen and blood lymphocyte populations, single cells suspensions were incubated in ACK lysis buffer for 5 mins to lyse red blood cells (RBCs). 1x10^6^ splenocytes or blood immune cells were then added to each flow tube. Fc block was added before antibody staining for 20 mins at 4°C. Samples were then washed twice with flow cytometry buffer (PBS + 2% FBS) and acquired on a CytoFLEX 30 (Beckman Coulter). Data were analyzed with CytExpert software. NK and T cell functional measurements were performed using fresh blood or spleen lymphocytes that were cultured with PMA/ionomycin for 4h in the presence of brefeldin A (1μL/mL) at 37°C. Following this, cells were washed twice with PBS, and then stained for NK/T cell markers. BD Cytofix/Cytoperm kit was used to fix and permeabilize cells using the manufacturer’s protocol. Intracellular staining for granzyme B and IFNγ was performed. All antibodies used are listed in **Supplementary Table S1**.

### 2.6 Immunogenic cell death assays

We obtained conditioned media (CM) by culturing 5x10^5^ cells in 24-well plates for 24h followed by treatment with ICD inducers at their IC50 doses or infection with VSVd51 at 10 MOI for 24h. *Flow cytometry:* treated or infected cells were processed as described above and stained as described in the flow cytometry section. Antibodies are listed in **Supplementary Table S1**. Bioimaging was performed using a fluorescence microscope (Leica). *Western blot:* proteins from cell-free CM (HMGB1) were resolved by SDS-PAGE and transferred to Immun-Blot-PVDF membranes (BioRad) for immunoblotting. Protein expression was detected using specific primary antibodies (1:1000) and corresponding HRP-conjugated secondary antibodies (1:10000). Protein expression was visualized by chemiluminescence detection (ChemiDoc, BioRad). Antibodies are listed in **Supplementary Table S1**. For Adenosine 5′-triphosphate *(ATP) detection*, the relative luminscent unit (RLU) of ATP in the CM was measured with the ENLITEN-ATP kit (Promega). Briefly, 100 μl of CM were transferred to 96-well opaque plates, 100µl of reconstituted rLuciferase/Luciferin reagent was added to each well followed by measurement of luciferase using a luminescence microplate reader (Fusion 3.0).

### 2.7 ELISAs

Culture supernatants were diluted 5-fold. ELISA kits for detecting TNFα, IL-10, IL-1β, CCL2, CCL4, CCl5 (all Peprotech), TGFβ Elisa (Invitrogen), PGE2 (Cayman), IFN1β (R&D Systems) were performed according to manufacturer’s instructions.

### 2.8 Spheroid and co-culture assay

2.5x10^4^ TNBC cells were suspended in 100% Matrigel (Corning) in a 48-well plate, and 300μl of the corresponding media were added over the spheroid plugs in each well. For co-culture assays with immune cells, the human spheroids were resuspended in 65-70% of Matrigel and co-cultured with human peripheral blood derived DCs, and 4T1 spheroids were resuspended in 65% of Matrigel and co-cultured with purified bone marrow dendritic cells (BMDCs). Human DCs or mouse BMDCs were labeled with CellTracker Deep Red Dye (ThermoFisher) at a final concentration of 1X in 1ml of complete media. The labeled cells were incubated for 30 minutes at 37˚C. Cells were washed (15,000 RPM 10 minutes at room temperature). The labelled cells were resuspended in complete media, counted, and co-cultured with treated spheroids (1 spheroid:10 immune cells).

### 2.9 Bone marrow isolation and differentiation to DCs

The tibias and femurs were removed and processed from euthanized BALB/c mice. The two ends of the bones were cut, and bone marrow was flushed out by washing with cold 1X PBS using an insulin syringe. Following filtration (70 μm) and washing to remove tissue debris, the resulting bone marrow cells were resuspended in DC media (complete RPMI supplemented with recombinant mouse IL-4 (4.5 ng/ml) and recombinant mouse GMCSF (5ng/ml) (Peprotech)). 1x10^6^ cells were seeded in 10 cm culture plates. On day 3, 1 ml of DC media was added. On days 5-7, all floating cells were harvested and washed with 1X PBS. The supernatant was removed, and the pellet was resuspended in DC media, counted, and co-cultured with the CM of treated/infected 4T1 cells for 48 hours at 37°C. Following incubation, cells were washed, and flow cytometry was performed for analyzing DC maturation markers (CD11c, MHCII, CD80, CD86).

### 2.10 Quantitative PCR of mouse BMDCs

1.5x10^6^ DCs were seeded in six-well plates. The CM of 4T1 cells (1.5x10^5^) after *in vitro* treatment with FLU, Oxa (IC50 concentrations), CM of 4T1 infected with VSVd51, or CM of non-treated 4T1 cells were added to the DCs cultures. Twenty-four hours post-treatment, DCs were harvested, and total RNA was isolated by Trizol (Invitrogen) according to the manufacturer’s instructions. qPCR was performed using RNA pooled from two independent experiments. RNA was then used for reverse transcription and qPCR was performed, validated and analyzed by the RNomics Platform at the Université de Sherbrooke (Bio-Rad CFS RealTime system) according to the protocols previously established by Hellemans et al. (31) and Vandesompele et al. (32). Primers’ sequences are available in **Supplementary Table S2**. RNA integrity was assessed with an Agilent 2100 Bioanalyzer (Agilent Technologies). Results were represented as fold change of DCs co-cultured with CM of treated samples relative to DCs co-cultured with the CM of non-treated cells.

### 2.11 Phagocytosis assay

1.5x10^5^ 4T1 cells were treated with ICD inducers and plated in a 48 well plate. Twenty-four hours post-treatment, the treated cells were harvested and labeled with Bodipy TMR (Invitrogen #D6117) at a final concentration of 2mM for 30 mins. The labeled cells were washed 3 times and further co-cultured with labeled-BMDCs as described above, then added to the cell cultures and incubated overnight. The ratio of 4T1 cells to BMDCs was 1:5. Twenty-four hours after co-culture, the labeled tumor cells and BMDCs were assessed by flow cytometry. Percentage of phagocytosis was calculated according to the formula: (number of the double-positive population of labeled BMDCs and 4T1 treated tumors) *100/number of labeled DCs). In addition, the live imaging of labeled co-culture cells was performed by CellDiscover7 microscopy, and the final images were analyzed by Zenblue software. The merged images representing the 4T1 tumors engulfed by DCs were calculated by Image J software, representing the number of phagocytic DCs engulfing the treated 4T1 cells.

### 2.12 Human polarization and migration assays

*Polarization:* Human monocytes were isolated from peripheral human blood using the Human CD14^+^ isolation kit (Stemcell). 5x10^5^ monocytes were seeded in 24-well plates in cRPMI and incubated overnight at 37°C and 5% CO2. 24h later, the monocyte media was replaced with the CM from infected human cell lines. Undifferentiated monocytes remained in complete media as M0; LPS (50ng/ml) (Millipore Sigma) and recombinant human IFNγ (20ng/ml) (BioBasic Inc) were added to monocyte cultures for differentiation into M1-like macrophages; recombinant human IL-10, IL-4, and TGFβ (BioBasic Inc) all at a final concentration of 20ng/ml were added to monocyte cultures for differentiation to M2-like macrophages. Monocytes were harvested and processed for flow cytometry as described above following 18h culture. *Migration:* 200μl of CM were placed in the lower well of Boyden chambers, separated by a 5 mm-pore polycarbonic membrane (Neuro Probe). 6x10^5^ human PBMC were added to the top chamber, followed by incubation at 37°C, 5% CO2 for 45 mins. Following this, the media in the bottom chamber was harvest and quantification of migrated cells by Trypan Blue exclusion was performed. The cells were stained and acquired by flow cytometry as described above. Antibodies are listed in **Supplementary Table S1**.

### 2.13 Statistical analysis

All statistical analyses were conducted using Prism 7 (GraphPad). Unpaired two-tailed t tests were used for comparing uninfected or infected cells or differentially treated mice. Survival differences of tumor-bearing and treated mice were assessed using Kaplan–Meier curves and analyzed by log-rank testing. P<0.05 was considered as statistically significant.

## 3 Results

### 3.1 Immunogenic cell death of mouse and human TNBC cells can be induced following *in vitro* treatment with the chemotherapeutic agent oxaliplatin and the seasonal influenza vaccine

**Figure 1:**
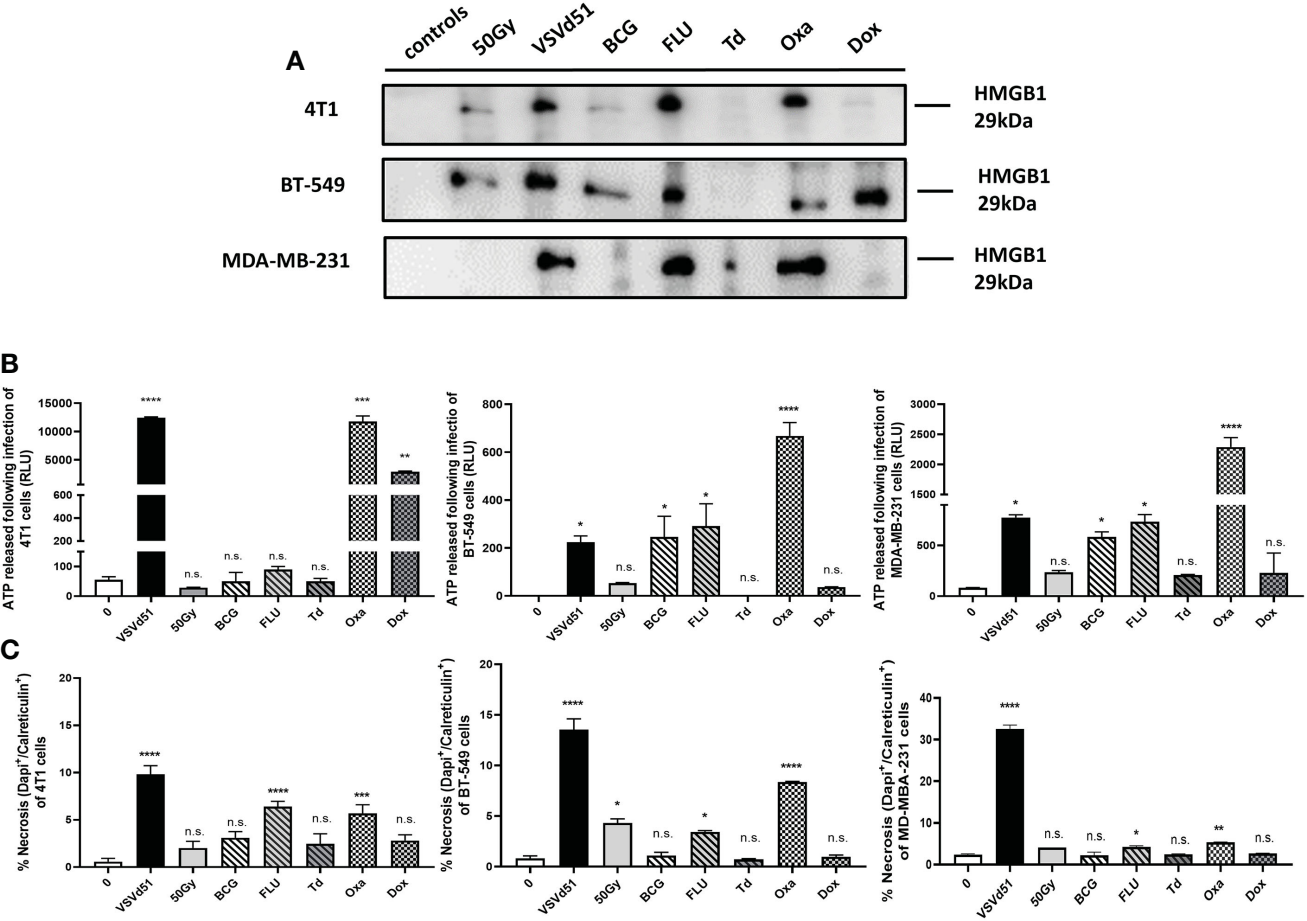
Immunogenic cell death of mouse and human TNBC cells can be induced following *in vitro* treatment with the chemotherapeutic agent oxaliplatin and the season influenza vaccine. **(A)** Western blot analysis of HMGB1 from cell-free supernatants, **(B)** luminometry measurement of relative ATP from cell-free supernatants, and **(C)** measurement of cell surface calreticulin of TNBC cell lines treated with ICD inducers at their IC50 concentrations or infected with VSVd51 at 10 MOI for 24h. The results were compared to non-treated cells. All data are representative of at least three similar experiments where n=3 for technical replicates, *P < 0.05; **P < 0.01; ***P < 0.001; ****P < 0.0001; (n.s., no significance).

We have previously reported that TNBC cells exhibit a necrotic cell death phenotype after infection with VSVd51. Moreover, our work highlighted that VSVd51 infection-induced necrosis was immunogenic in nature due to the release of DAMPs and other soluble mediators critical to the formation of anti-tumor immune responses (17). To further improve the efficacy of the ICV approach, we tested other immune modulators that could be used to formulate a more immunogenic prime vaccine. Accordingly, we treated human (BT-549 and MDA-MB-231) and mouse (4T1) TNBC cell lines with different classes of ICD inducers, including chemotherapeutic agents such as oxaliplatin (Oxa) and doxorubicin (Dox) widely used as standard of care chemotherapy for TNBC; and commercially available vaccines against infectious diseases that could provide an adjuvant effect, including the seasonal flu vaccine (FLU), Bacillus Calmette Guerin (BCG) vaccine against tuberculosis, and Td adsorbed vaccines against tetanus-diphtheria. Following treatment of TNBC cells with IC50 doses of ICD inducers (**Supplementary Figure S1**), we measured the resulting levels ICD biomarkers, including HMGB1 (**Figure 1A**) and ATP release (**Figure 1B**) and cell surface CRT exposure (**Figure 1C**). Among chemotherapeutic ICD inducers, Oxa treated-4T1 and MDA-MB-231 cells released higher levels of HMGB1 compared to Dox treatment and non-treated controls. The release of HMGB1 was not significant when comparing Oxa and Dox treatment for BT-549 cells. However, HMGB1 release was significantly higher following Dox treatment when compared to non-treated BT-549 cells. Treatment of TNBC cells with FLU resulted in the release of higher levels of HMGB1 for all treated cells compared to BCG and Td adsorbed (**Figure 1A**). ATP release was significantly higher after treatment of TNBC cells with Oxa compared to all other ICD inducers (**Figure 1B**). For CRT exposure, Oxa and FLU treated TNBC cells resulted in their highest cell surface level expression (**Figure 1C**). Taken together, the release of HMGB1, ATP and CRT exposure were the highest following treatment of TNBC cells with Oxa and FLU, among ICD inducers tested.

### 3.2 ICD inducers improve the release of immunogenic mediators from TNBC cells

**Figure 2:**
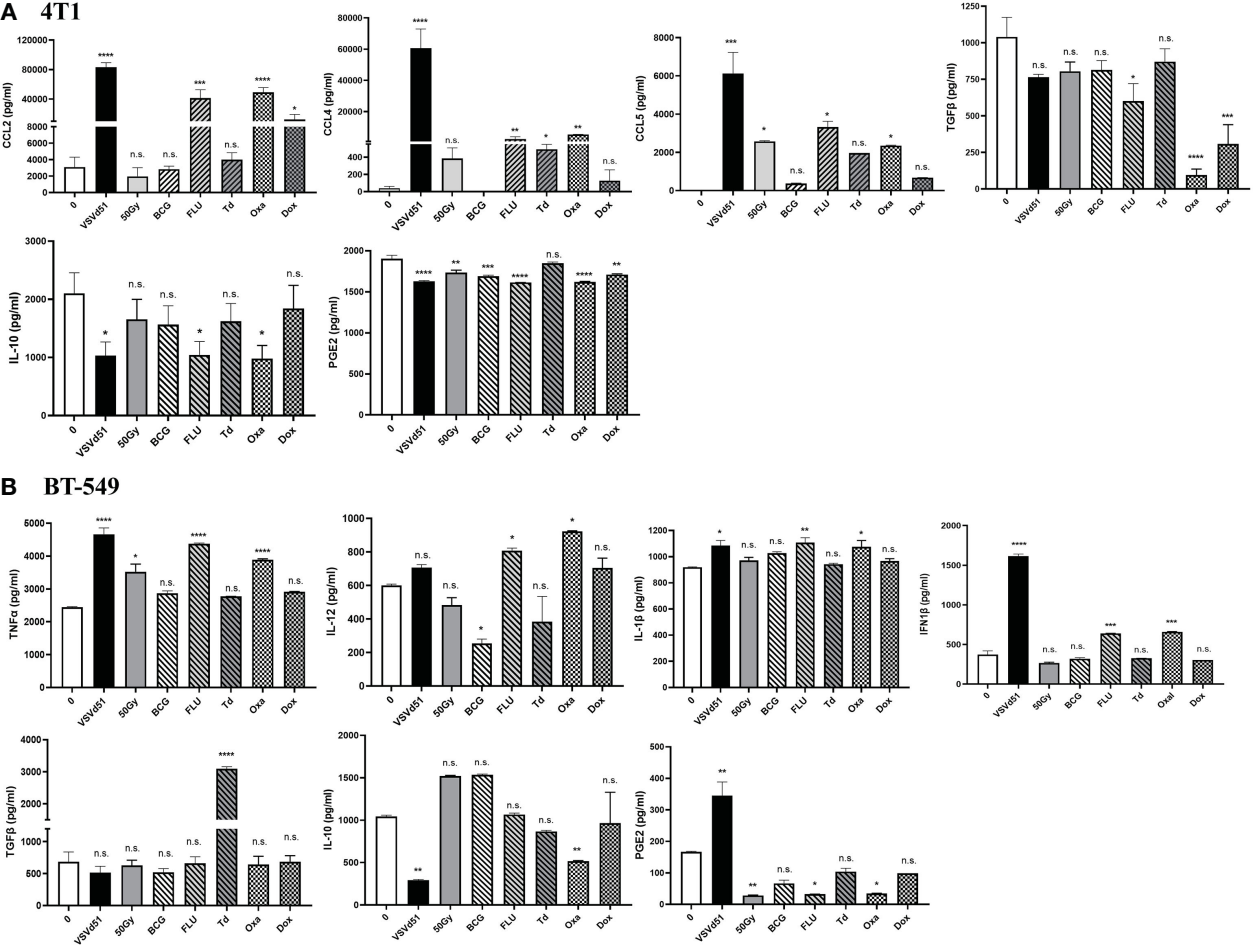
ICD inducers improve the release of immunogenic mediators from TNBC cells. Cytokine and chemokine levels from **(A)** mouse 4T1 and human **(B)** BT-549 cell line culture supernatants were quantified by ELISA following treatment of with IC50 concentrations of ICD inducers or infected with 10 MOI of VSVd51 for 24h. The results were compared to non-treated cells. ELISA was performed using supernatant pooled from 3 independent experiments, where *P < 0.05; **P < 0.01; ***P < 0.001; ****P < 0.0001; (n.s., no significance).

Next, we sought to determine if other critical factors such as pro- and anti-inflammatory cytokines and chemokines that modulates the TME in addition to DAMPs are released following *in vitro* treatment of TNBC cells with ICD inducers. We detected enhanced concentrations of pro-inflammatory cytokines, including CCL2, CCL4, and CCL5 released from 4T1 cells following their treatment with Oxa and FLU for 24h. Treatment of 4T1 cells with Dox and Td also raised the levels of these inflammatory cytokines compared to non-treated cells, but their levels were not as high compared to Oxa and FLU treatment (**Figure 2A**). In contrast, treatment of 4T1 mouse cells with Oxa and FLU diminished the amounts of anti-inflammatory/immune suppressive cytokines IL-10, TGFβ and PGE2 compared to untreated cells. In parallel, the highest concentrations of pro-inflammatory cytokines, including IFN1β, TNFα, IL-12, and IL-1β, were detected after treatment of human BT-549 and MDA-MB-231 cells with Oxa and FLU, among other ICD inducers tested (**Figure 2B**; **Supplemental Figure S2**). Similar to the 4T1 data, the immune suppressive cytokine PGE2 was detected at lower levels following treatment of both human TNBC cell lines with the FLU vaccine compared to untreated cells. Reduced IL-10 levels were also detected following Oxa treatment of both human TNBC cell lines. Taken together, our results suggest that a combination of inflammatory and immune suppressive signals contribute to TNBC cell line immunogenicity following treatment with ICD inducers. Further, these results reveal that Oxa and FLU provoke the release of more pro-inflammatory cytokines, while simultaneously decreasing anti-inflammatory signatures compared to other prime-vaccine immune modulators.

### 3.3 Enhanced recruitment and activation of mouse dendritic cells following exposure to TNBC spheroids treated with oxaliplatin or influenza vaccine

**Figure 3:**
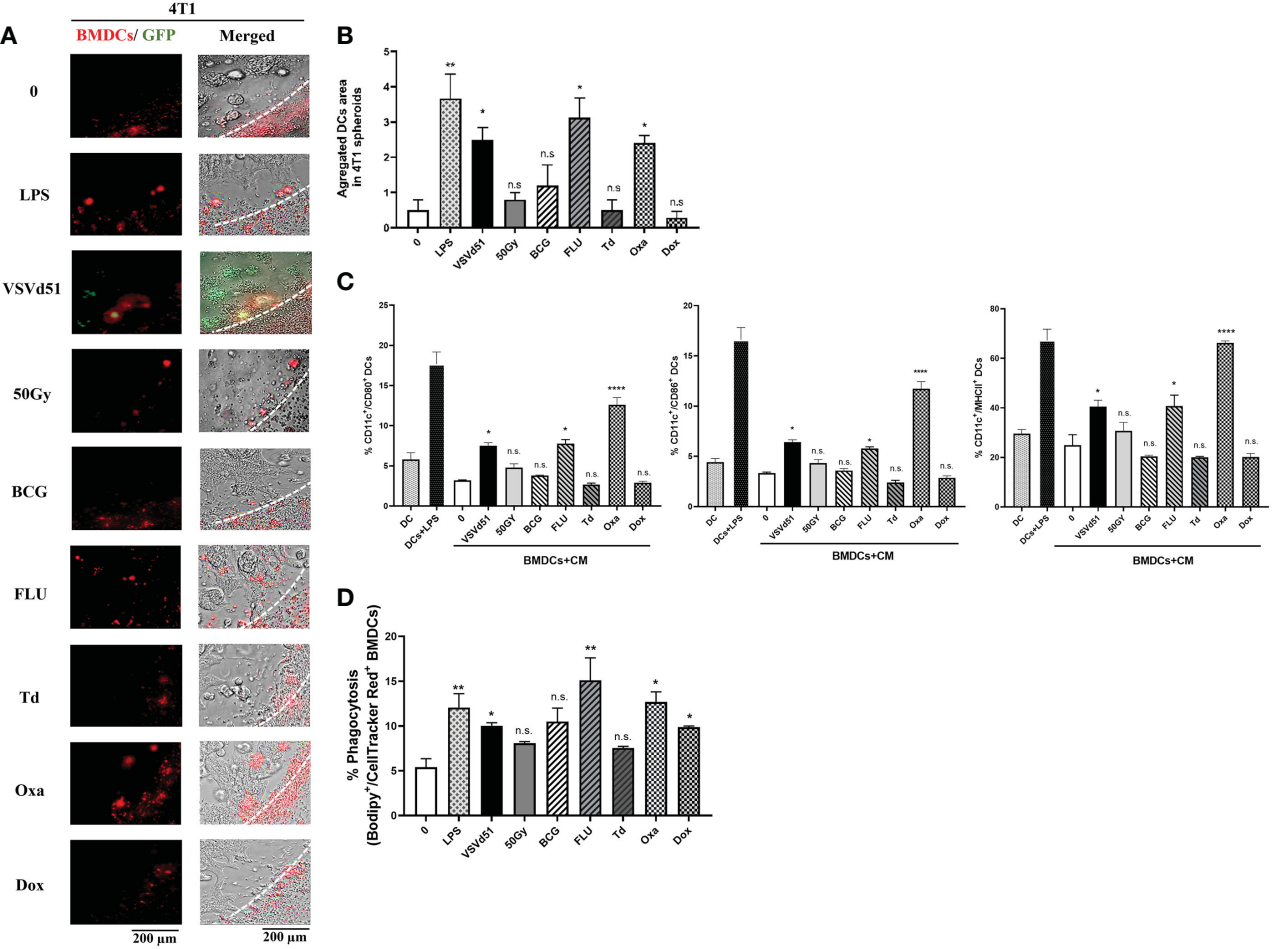
Enhanced recruitment and activation of mouse dendritic cells following exposure to TNBC spheroids treated with oxaliplatin or influenza vaccine. Live cell images from **(A)** 4T1 spheroids co-cultured with deep red CellTracker-labelled BMDCs, 24h following their treatment with ICD inducers at IC50 concentrations. LPS (1μg/ml) treated spheroids were used as positive controls. **(B)** Quantification of microscopy images representing the number of infiltrated and aggregated immune cells around treated spheroids. Dashed lines delineate the edge of spheroids. The results are compared to non-treated cells. **(C)** Flow cytometry analysis of maturation markers of BMDCs following 48h of exposure to CM of 4T1 cells treated with indicated ICD inducer at IC50 concentration or 10 MOI of VSVd51. LPS treated (1μg/ml) BMDCs were used as a positive control. **(D)** Flow cytometry analysis of phagocytic BMDCs labelled with deep red CellTracker and co-cultured with Bodipy TMR labelled 4T1 cells. All data are representative of at least three similar experiments where n=3 for technical replicates, *P < 0.05; **P < 0.01; ****P < 0.0001; (n.s., no significance).

DAMPs and cytokines are essential signals for attracting immune cells to the site of stimulation/infection leading to the maturation of DCs and activation of CD8^+^ T and NK cells. Therefore, we explored whether the secreted soluble mediators described above are critical for attracting TNBC-targeted immune cells. To do so, we performed live imaging of a co-culture system, including mouse bone marrow derived DC (BMDC) with 4T1 spheroids. We grew the 4T1 cell line as spheroids to better recapitulate the glandular structures of breast cancer cells *in vivo*. Following the migration of BMDCs towards the spheroids, we quantified areas of infiltrated and aggregated DCs. Our data illustrates that spheroids pretreated with Oxa or FLU recruit more BMDCs than other treatments (**Figures 3A, B**). Furthermore, we were interested in assessing whether these BMDCs are more mature. Accordingly, we measured cell surface maturation markers and observed that those BMDCs exposed to the CM of 4T1 spheroids treated with Oxa and FLU exhibited a more mature (CD11c^+^/CD80^+^/CD86^+^/MHCII^+^) cell surface phenotype (**Figure 3C**). Moreover, we evaluated a panel of immunogenic and tolerogenic gene signatures from these BMDCs following co-culture with CM. Our qPCR data revealed that the CM of 4T1 spheroids following treatment with Oxa and FLU skewed the BMDCs toward an immunogenic phenotype confirmed by elevated mRNA expression of the MHCI gene H2-k1, the canonical DC co-stimulatory receptor CD40, the T-cell polarizing cytokines TNFα, and IL-2 (**Supplemental Figure S3**). In contrast, the gene signature associated with a tolerogenic phenotype, including gene expression of CTLA-4, IL-10, and IDO on BMDCs co-cultured with CM of 4T1 cells treated with FLU and Oxa were down-regulated compared to BMDCs co-cultured with CM of non-treated 4T1 cells (**Supplemental Figure S3**). Lastly, to confirm whether BMDCs can uptake tumor antigens more efficiently after co-culturing with ICD treated 4T1 cells, we performed a phagocytosis assay. Previously treated and Bodipy-labeled 4T1 cells were co-cultured with cellTracker red-labeled BMDCs. The phagocytic capacity of BMDCs to engulf tumor cells was then assessed by flow cytometry. We detected higher percentages of double positive BMDCs in co-culture with 4T1 cells treated with Oxa and FLU, suggesting a higher phagocytic ability of these DCs (**Figure 3D**). Taken together, these results suggest the heightened immunogenicity of Oxa and FLU treated 4T1 cells increased their capacity to recruit and activate critical functions of DCs, that are key players in the generation of anti-tumor immune responses.

### 3.4 Increased migration and functionality of human DC and effector immune cells following co-culture with oxaliplatin and influenza vaccine treated human TNBC spheroids

**Figure 4:**
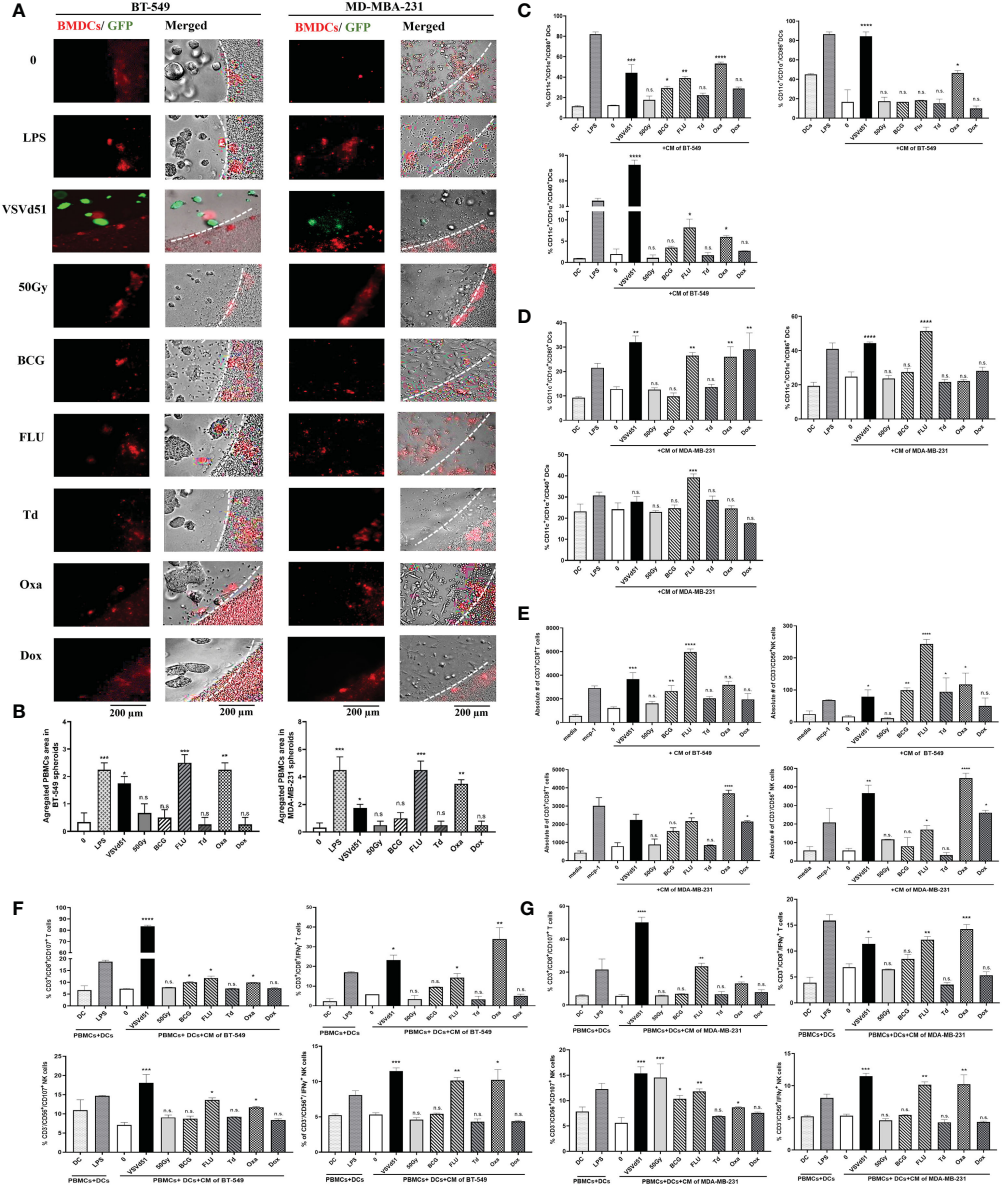
Increased migration and functionality of human DC and effector immune cells following co-culture with oxaliplatin and influenza vaccine treated human TNBC spheroids. Live cell images from **(A)** BT-549 and MD-MBA-231 spheroids co-cultured with deep red CellTracker-labelled human DCs, 24h following their treatment with ICD inducers at IC50 concentrations. LPS (1μg/ml) treated spheroids were used as positive controls. **(B)** Quantification of microscopy images representing the number of infiltrated and aggregated immune cells around the treated spheroids. Dashed lines delineate the edge of spheroids. The results are compared to non-treated cells. Flow cytometry analysis of maturation markers on human DCs following 48h of exposure to CM of **(C)** BT-549 or **(D)** MDA-MB-231 cells treated with indicated ICD inducers at IC50 concentration or 10 MOI of VSVd51. LPS treated (1μg/ml) human DCs are used as a positive control. **(E)** Flow cytometry quantification of migrated purified human CD3^+^/CD8^+^ T cells and CD3^-^/CD56^+^ NK cells towards CM from BT-549 and MDA-MB-231 cells incubated with indicated treatments; MCP-1 (50ng/ml) was used as a positive control for immune cell migration. Flow cytometry analysis of purified human CD3^+^/CD8^+^ T cells and CD3^-^/CD56^+^ NK cells in tri-cultures with human DCs previously exposed to CM from **(F)** BT-549 or **(G)** MDA-MB-231 treated spheroids with indicated treatments. The results are compared to non-treated cells. All data are representative of at least three similar experiments where n=3 for technical replicates, *P < 0.05; **P < 0.01; ***P < 0.001; ****P < 0.0001; (n.s., no significance).

To evaluate whether these results in mouse cell lines and immune cells can be recapitulated in human cells, we isolated human DCs from peripheral blood and cultured them with spheroids grown from human BT-549 and MDA-MB-231 TNBC cell lines. First, we measured the migration of human DCs towards the spheroids, and observed higher infiltration and aggregates of DCs towards spheroids treated with Oxa or FLU (**Figures 4A, B**). Next, we measured cell surface maturation/differentiation markers and observed that those DCs cultured with spheroids treated with Oxa or FLU demonstrated a more mature (CD1α^+^/CD11c^+^/CD80^+^/CD86^+^/CD40^+^) cell surface phenotype (**Figures 4C, D**). Following the characterization of DCs, we assessed effector immune cell migration and functionality. We performed a migration assay using purified human CD8^+^T cells and NK cells in co-culture with CM from ICD-treated human TNBC spheroids. We observed an increased migration of CD8^+^T cells and NK cells toward the CM of BT-549 and MDA-MB-231 spheroids treated with Oxa or FLU, compared to non-treated cells. Importantly, the CM from Oxa and FLU treated spheroids induced the highest amount of effector cell recruitment compared to other prime vaccine candidates (**Figure 4E**). In a tri-culture assay comprising of purified DCs, TNBC spheroids, and either purified CD8^+^T cells or NK cells, heightened levels of IFNγ^+^ and CD107^+^ CD8^+^T and NK cells were detected following Oxa or Flu treatments (**Figures 4F, G**). These results suggest that human DCs stimulated by Oxa-treated or FLU-treated TNBC spheroids can attract and activate effector immune cells that have the potential of recognizing and eliminating metastatic human TNBC tumors.

### 3.5 Early surgery coupled with heterologous Prime boost vaccination improves survival in the BALB/c-4T1 model of TNBC

**Figure 5:**
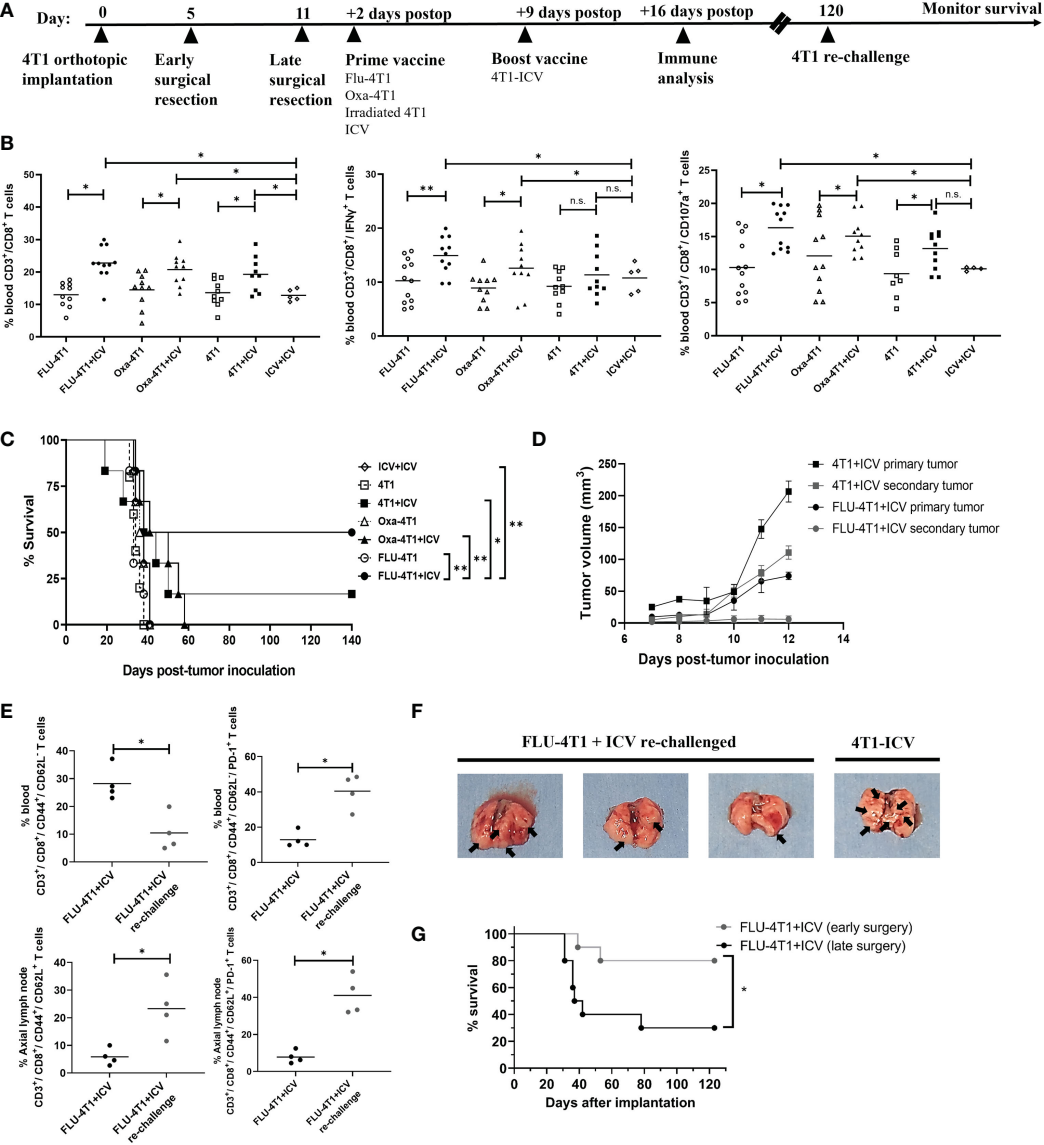
Heterologous prime-boost vaccination improves survival in the BALB/c-4T1 model of TNBC. **(A)** Timeline of *in vivo* BALB/c-4T1 experiment. BALB/c mice were orthotopically implanted with 1x10^5^ 4T1 cells followed by a complete primary tumor resection on indicated days. Two days postoperatively, mice received 1 dose of the prime vaccine in the cleared mammary fat pad (FLU-4T1, Oxa-4T1, irradiated 4T1, ICV). Nine days postoperatively, mice received their 4T1-ICV boost vaccine. Immune functional analyses, re-challenge and monitoring were performed as indicated. **(B)** Immune cell suspensions from the peripheral blood of mice following indicated treatments were stained with T cell markers (CD3^+^, CD8^+^, IFNγ^+^, CD107a^+^) and analyzed by flow cytometry. **(C)** Kaplan-Meier survival analysis of mice receiving prime-boost ICV. n=10-12 mice/group. *P < 0.05; **P < 0.01; (n.s., no significance), log-rank test. **(D)** Tumor growth measurements comparing re-challenged 4T1 tumors with their corresponding primary tumor from 4T1-ICV and FLU-4T1-ICV treatment cohorts. **(E)** Flow cytometry analysis of central memory T cells in the axillary lymph node and effector memory T cells in the blood of FLU-4T1+ICV treated cohort before and after re-challenging with 4T1 tumors. **(F)** Representative lung pictures from FLU-4T1+ICV re-challenged and 4T1-ICV treated cohorts. **(G)** Kaplan-Meier survival analysis of mice receiving early vs. late surgery and prime boost ICV. n=10-12 mice/group. *P < 0.05; **P < 0.01; (n.s., no significance), log-rank test. All flow cytometry data are representative of three similar experiments where n=4 mice/treatment, *P < 0.05; **P < 0.01; (n.s., no significance).

To corroborate these *in vitro* results with *in vivo* data, we administered the top prime vaccines followed by a boost ICV in BALB/c mice bearing orthotopic 4T1 tumors after primary tumor resection (**Figure 5A**, timeline). This model makes use of an aggressive mouse stage IV TNBC from the BALB/c strain that spontaneously metastasizes from the mammary glands to multiple distant sites, in particular the lungs. Primary tumor resection is performed to prolong survival due to the fast-growing primary tumor and to provide a therapeutic window for adjuvant treatment. Since we observed that Oxa or FLU treated 4T1 cells were the top inducers of ICD for DC maturation, phagocytosis, and effector cell function, we included the following treatment cohorts: prime vaccine alone (irradiated 4T1 cells, Oxa-4T1, FLU-4T1); and prime and boost vaccine (4T1+ICV, Oxa-4T1+ICV, FLU-4T1+ICV). Importantly, a control group consisting of an ICV prime followed by the same ICV boost vaccine was included (i.e., homologous vaccination, ICV+ICV). We observed that postoperative administration of a heterologous prime-boost vaccine, regardless of the ICD inducer, significantly improved CD3^+^/CD8^+^ T cells proportions and functionality (IFNγ^+^, CD107^+^ degranulation) compared to the administration of prime vaccine alone or homologous vaccination (ICV+ICV) (**Figure 5B**). Notably, we detected the longest survival in mice that received a prime vaccine consisting of FLU-treated 4T1 cells followed by an ICV boost (**Figure 5C**).

Following this, we sought to investigate whether these long-term survivors harbor immunological memory against 4T1 tumors. Therefore, we re-challenged surviving mice (FLU-4T1+ICV; 4T1+ICV cohorts) by implanting 4T1 tumors in the opposite mammary fat pad at 120 days post primary tumor implantation. Following 4T1 tumor re-challenge, FLU-4T1+ICV cohorts were completely protected from the development of secondary tumors (**Figure 5D**) compared to previously measured fast growing primary tumors following initial tumor implantation. In contrast, we observed slower secondary tumor growth in 4T1+ICV vaccinated cohorts. Importantly, we measured higher percentage of effector circulating memory T cells (CD3^+^, CD8^+^, CD44^+^, CD62L^-^) and central memory T cells (CD3^+^, CD8^+^, CD44^+^, CD62L^+^) in the tumor draining lymph nodes of FLU-4T1+ICV mice following tumor re-challenge (**Figure 5E**). The resected lungs from re-challenged mice also contained less metastatic nodules compared to the lungs of mice treated with 4T1-ICV (**Figure 5F**). These findings suggest the presence of tumor-specific immune memory against 4T1 tumors in mice receiving heterologous prime-boost vaccination.

Even though the heterologous prime-boost vaccine strategy resulted in improved survival for treated mice, we were dissatisfied with the overall survival percentages. As previously published, the 4T1 cell line is an extremely aggressive stage IV TNBC mouse tumor that spontaneously metastasizes to the brain, lungs, liver and bones. In unpublished observations, we noticed metastatic colonization in the lungs as early as day 5 following orthotopic breast tumor implantation. We reasoned that earlier primary tumor resection may reduce lung metastases and prolong survival. In our established model, we typically performed primary tumor resection at day 10-12 following tumor implantation when a large tumor (75-100mm^3^) is observed. However, 20-50mm^3^ size tumors are evident in all 4T1 implanted mice by day 5. Therefore, we repeated the heterologous prime (FLU-4T1) and boost (ICV) vaccine regimen comparing either early surgical resection of primary tumors at day 5 or later surgery at day 11. We observed that the combination of early surgical resection combined with heterologous prime-boost vaccination resulted in significantly improved survival of mice bearing aggressive 4T1 orthotopic tumors (**Figure 5G**). Taken together, these preclinical results suggest that therapeutic cancer vaccines can be combined with early surgical intervention to improve response in aggressive TNBC.

## 4 Discussion

Poor prognosis TNBC patients who fail neoadjuvant chemotherapy and surgery urgently need effective therapies to prevent recurrence and progressive disease. Multiple recent efforts using checkpoint inhibitor monotherapy and in combination with chemo- and targeted-therapies have been made to improve outcome for TNBC patients. A significantly improved progression free survival (PFS) and a positive median overall survival (OS) was observed in TNBC patients receiving αPD-L1 atezolizumab with nab-paclitaxel, compared to patients receiving nab-paclitaxel and placebo in the phase III Impassion 130 trial (22, 33). The phase Ib/II KEYNOTE-150 trial investigating the combination of αPD-1 pembrolizumab with eribulin demonstrated benefits in PFS and OS in preliminary findings (22, 34). Despite these new immunotherapies, early results show modest improvements in survival and only in subsets of TNBC patients. This underscores the need to continue the development of novel immunotherapies for broader subsets of TNBC patients. Given the heterogeneous and immunogenic nature of TNBC, we reasoned that the development of personalized therapeutic vaccines is warranted for poor prognosis TNBC.

Autologous tumor cell vaccines are an antigen agnostic form of personalized immunotherapy that eliminates the need to sequence the tumor prior to vaccine formulation, saving both precious time and money. Treatment with autologous tumor cells will reveal a patient’s own complete and individualized tumor antigen repertoire, thus lessoning the chances of heterogenous tumor immune evasion (18–20). A major limitation of whole tumor cell-based vaccines is the lack of immunogenicity of self-antigens. A treatment approach combining cytokines and whole tumor cells could significantly delay tumor progression. This has been shown to involve the development of a pro-inflammatory microenvironment that promotes immune system activation against TAAs (35). However, the majority of patients treated with this combination do not respond. This could be due to the lack of immunogenicity of the tumor cell vaccine and cytokine combination or profound cancer-induced immune suppression. The FANG vaccine was developed to address these limitations. It is composed of autologous tumor cells transfected with an immunogenic GM-CSF/shRNAi furin vector (36). Remarkably, stage III/IV ovarian cancer patients treated with the FANG vaccine demonstrated extended recurrence-free survival, likely due to high levels of T cell activation (37). These findings reveal the promising clinical potential of immunogenic autologous tumor vaccines.

We and others are working to improve the whole cell vaccination strategy by infecting tumor cells *ex vivo* with OV (17, 21, 24, 25, 38). As proof of concept for TNBC, we recently demonstrated that the intratumoral delivery of postoperative autologous TNBC cells infected with VSVd51, provided a significant therapeutic benefit to aggressive mouse models of TNBC (17). Both NK and CD8^+^ T cells demonstrated enhanced cytokine secretion and cytotoxicity following VSVd51-ICV administration (17). Moreover, we observed improved survival of mice when we combined ICV with αPD-1 checkpoint inhibitor therapy. We sought to further enhance the therapeutic efficacy of ICV by applying a heterologous prime-boost vaccination approach where irradiated whole tumor cells are delivered twice to focus the immune response on 4T1 specific TAA. In the prime vaccine, the 4T1 cells are modulated with immune adjuvants, while the boost vaccine involved infection of 4T1 cells with VSVd51 (**Figure 5B**). Using several classes of known ICD inducers, including chemotherapeutic agents and existing infectious disease vaccines, we determined that *in vitro* treatment of both mouse and human TNBC cell lines with oxaliplatin or the seasonal influenza vaccine resulted in the highest levels of ICD biomarker exposure, including HMGB1, ATP and CRT. Furthermore, critical soluble mediators known to be involved in immune cell recruitment and activation such as CCL2, CCL4, CCL5, TNFα, IFN1β, and IL-12 were detected at significantly higher levels in the CM of Oxa and FLU treated TNBC cell lines. We demonstrated that these biomarkers of ICD and proinflammatory cytokines attracted and differentiated both mouse BMDCs and human DCs. Accordingly, an immunogenic gene signature was detected in mouse BMDCs, exhibiting elevated levels of MHCI, CD40, TNFα and IL-12; and dampened levels of IL10, IDO1 and CTLA4. Subsequently, increased migration and activation of both human and mouse effector CD8+ T and NK cells were detected. From *in vivo* experiments, we observed that postoperative prime-boost vaccination with FLU treated 4T1 cells followed by VSVd51-ICV significantly improved initial and memory immune cell responses and provided durable survival benefits. Furthermore, we showed that VSVd51 served to further improve the booster vaccine efficacy when combined with early primary tumor resection.

Surgical resection, especially of early stage disease is a critical intervention and provides a chance of cure for patients with cancer, breast cancer included. Resection of early stage breast disease results in less surgical complications and better outcome for the patient (39). The perioperative period has been characterized by an increased risk for accelerated growth of micrometastatic disease and enhanced formation of new metastatic foci. Perioperative factors including immunosuppression, anesthesia, hypothermia, and posteoperative complications may accompany later stage resections and contribute as potential deleterious factors contributing to worse outcome in patients (40). Our *in vivo* results demonstrated that early resection coupled with cancer vaccination translated to improved survival outcome compared to later surgery combined with the same cancer vaccine strategy.

**Figure 6:**
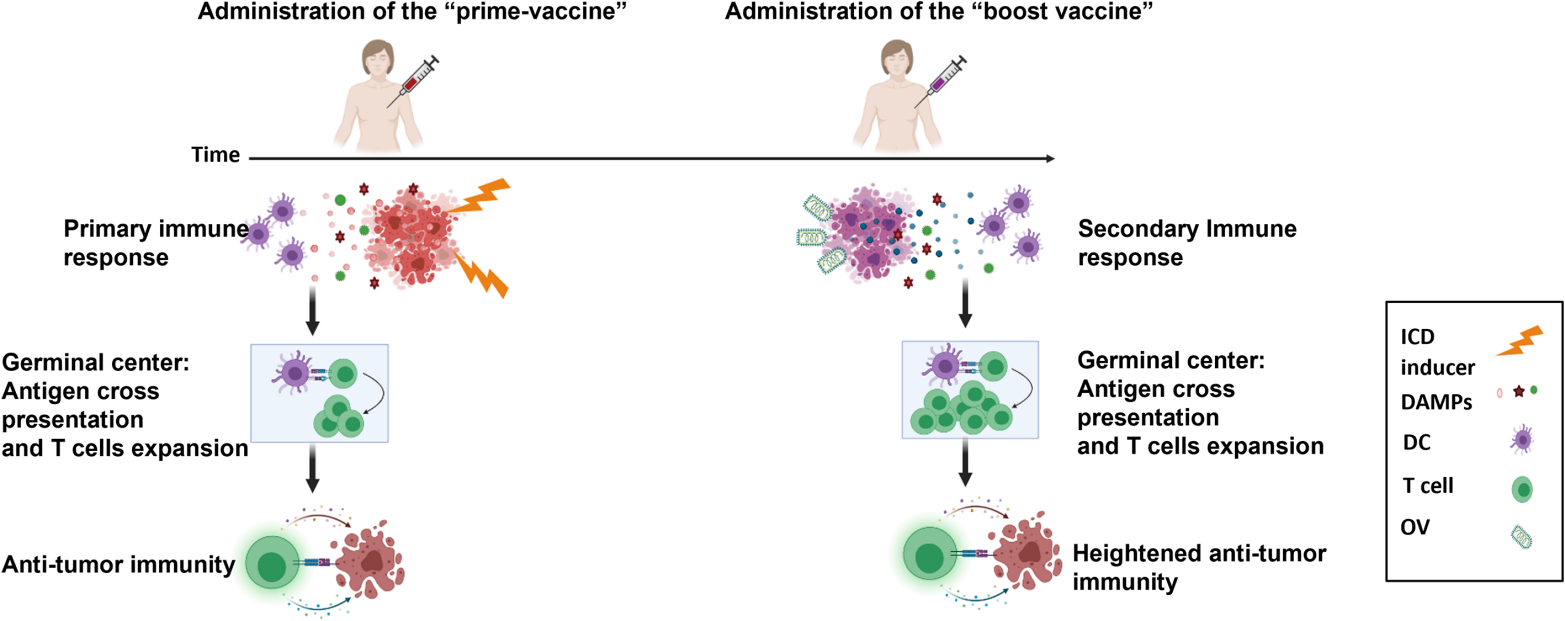
Model of heterologous prime-boost vaccination for TNBC. Adjuvant vaccination with a prime cellular vaccine results in the release of immunogenic cell death biomarkers (DAMPs, cytokines, chemokines) that recruit and activate antigen presenting cells to cross-present tumor associated antigens to tumor-targeted T cells. This is followed by the administration of a boost oncolytic virus-infected cellular vaccine to further focus the immune response on tumor antigens. An enhanced secondary immune response is instigated by this heterologous prime-boost vaccination to reduce metastatic and recurrent disease of TNBC.

We are working towards the design of a future clinical trial that consists of early primary tumor resection and an optimized heterologous prime-boost vaccination strategy to initiate ICD and TAA release to promote an antitumor immune response (**Figure 6**). Our *in vitro* and *in vivo* data showed that a superior immune and survival response is ultimately achieved through enhancing the prime vaccine with the seasonal flu vaccine. The established safety, efficacy and acceptability profile of the seasonal flu vaccine will accelerate its translation and potential incorporation into a therapeutic prime-boost cancer vaccine for TNBC. However, in the human setting, it is likely that many TNBC patients will have been previously vaccinated with a flu vaccine. This could potentially hinder or improve outcomes. The Zloza group demonstrated that intratumoral administration of an unadjuvanted seasonal flu vaccine reduced tumor growth, improved systemic antitumor immunity, and sensitized resistant tumors to immune checkpoint blockade immunotherapy in B16 experimental lung tumor models, whereas intramuscular injection did not. They also showed that previous influenza infection did not interfere with subsequent tumor reduction using the same heat-inactivated strain of influenza as a vaccine in the B16 model (41). Their results suggest that pre-existing immunity or immunization against flu antigens would not handicap the anti-tumor immune effect of the flu vaccine. Notably, their studies revealed that the anti-tumor effect is abrogated when they used an adjuvanted flu vaccine. Squalene-based emulsion adjuvants have been shown to induce strong innate immune pathways to generate strong B cell responses and antibody production against viral infections (41). Although we were able to detect anti-tumor immunity through the use an adjuvanted flu vaccine (Seqirus) in our studies, this could be due to differences in the overall formulation of our prime vaccine, which also included irradiated TNBC cells. In a separate study, the Masopust group similarly demonstrated that virus-specific memory T cells can be re-stimulated by viral peptides. These were injected intratumorally and resulted in reduced tumor growth, enhanced checkpoint blockade immunotherapy, and promoted survival in B16 tumor-bearing mice (42). We have not yet tried an unadjuvanted formulation in our prime vaccine nor have we attempted pre-vaccination of our mice before initiating our therapeutic model. These experiments to improve our prime boost cancer vaccine approach forms the basis of our ongoing studies.

Our prime boost vaccination strategy could be further followed by checkpoint inhibitor administration to sustain the anti-tumor activity of T cells at the tumor site. The Bell group recently showed that a neoadjuvant OV can be used to sensitize the tumor to checkpoint blockade therapy in preclinical TNBC models (43). However, these mouse models did not include surgical resection nor was the OV delivered as part of a cancer cell vaccine. For nonresectable disease, patient tissue obtained *via* biopsy could potentially provide source material for both the prime and boost vaccines. The clinical timing of prime-boost vaccine delivery in the context of frontline treatment for TNBC is a current focus of our investigations.

## 5 Conclusions

In summary, we characterized the mechanism and clinical potential of a heterologous prime-boost vaccination approach for the treatment of TNBC. We demonstrated that innate and adaptive immune cells play mediating roles in the *in vivo* efficacy of our heterologous vaccination strategy. These findings reveal the importance of effector and central memory subsets of cytotoxic T cells in the reduction of metastatic disease. Furthermore, our preclinical data demonstrate the potential of repurposing commercially available infectious disease vaccines, such as seasonal influenza vaccine as a strong ICD inducer to stimulate innate and adaptive immune cells. These vaccines have established safety data in many immune competent and comprised populations (infants, the elderly, pregnant individuals, HIV+ individuals, etc.) and could therefore translate very quickly to the clinics as a component of therapeutic cancer vaccines. In summary, our translational data demonstrate the potential of applying heterologous prime-boost vaccination for hard-to-treat cancers such as TNBC.

## Data availability statement

The raw data supporting the conclusions of this article will be made available by the authors, without undue reservation.

## Ethics statement

The animal study (protocol no. 2020-2606) was reviewed and approved by the Faculty of Medicine Animal Care Committee of the Universite de Sherbrooke.

## Author contributions

SN, CL, GS-C, HG and L-HT conducted experiments, read and reviewed the manuscript. SN, CL, HG and LD contributed towards the writing and critically revised the manuscript. L-HT conceived, designed and executed experiments, was a major contributor in writing the manuscript, and supervised the study. All authors contributed to the article and approved the submitted version.

## References

[1] Khosravi-Shahi P Cabezón-Gutiérrez L Custodio-Cabello S. Metastatic triple negative breast cancer: Optimizing treatment options, new and emerging targeted therapies. Asia Pac J Clin Oncol (2018) 14(1):32−9. doi: 10.1111/ajco.12748 28815913

[2] McAndrew N DeMichele A. Neoadjuvant chemotherapy considerations in triple-negative breast cancer. J Target Ther Cancer (2018) 7(1):52−69. 29577076 PMC5865448

[3] Lehmann BD Jovanović B Chen X Estrada MV Johnson KN Shyr Y. Refinement of triple-negative breast cancer molecular subtypes: Implications for neoadjuvant chemotherapy selection. PloS One (2016) 11(6):e0157368. doi: 10.1371/journal.pone.0157368 27310713 PMC4911051

[4] Adams S Gray RJ Demaria S Goldstein L Perez EA Shulman LN. Prognostic value of tumor-infiltrating lymphocytes in triple-negative breast cancers from two phase III randomized adjuvant breast cancer trials: ECOG 2197 and ECOG 1199. J Clin Oncol Off J Am Soc Clin Oncol (2014) 32(27):2959−66. doi: 10.1200/JCO.2013.55.0491 PMC4162494 25071121

[5] Loi S Michiels S Salgado R Sirtaine N Jose V Fumagalli D. Tumor infiltrating lymphocytes are prognostic in triple negative breast cancer and predictive for trastuzumab benefit in early breast cancer: Results from the FinHER trial. Ann Oncol Off J Eur Soc Med Oncol (2014) 25(8):1544−50. doi: 10.1093/annonc/mdu112 24608200

[6] Salgado R Denkert C Demaria S Sirtaine N Klauschen F Pruneri G. The evaluation of tumor-infiltrating lymphocytes (TILs) in breast cancer: Recommendations by an international TILs working group 2014. Ann Oncol Off J Eur Soc Med Oncol (2015) 26(2):259−71. doi: 10.1093/annonc/mdu450 PMC6267863 25214542

[7] Budczies J Bockmayr M Denkert C Klauschen F Lennerz JK Györffy B. Classical pathology and mutational load of breast cancer - integration of two worlds. J Pathol Clin Res (2015) 1(4):225−38. doi: 10.1002/cjp2.25 27499907 PMC4939893

[8] Banerji S Cibulskis K Rangel-Escareno C Brown KK Carter SL Frederick AM. Sequence analysis of mutations and translocations across breast cancer subtypes. Nature (2012) 486(7403):405−9. doi: 10.1038/nature11154 22722202 PMC4148686

[9] Karn T Jiang T Hatzis C Sänger N El-Balat A Rody A. Association between genomic metrics and immune infiltration in triple-negative breast cancer. JAMA Oncol (2017) 3(12):1707−11. doi: 10.1001/jamaoncol.2017.2140 28750120 PMC5824276

[10] Umansky V Sevko A. Tumor microenvironment and myeloid-derived suppressor cells. Cancer Microenviron Off J Int Cancer Microenviron Soc (2013) 6(2):169−77. doi: 10.1007/s12307-012-0126-7 PMC3717060 23242672

[11] O’Donnell JS Teng MW Smyth MJ. Cancer immunoediting and resistance to T cell-based immunotherapy. Nat Rev Clin Oncol (2019) 16(3):151−67. doi: 10.1038/s41571-018-0142-8 30523282

[12] Gruosso T Gigoux M Manem VSK Bertos N Zuo D Perlitch I. Spatially distinct tumor immune microenvironments stratify triple-negative breast cancers. J Clin Invest (2019) 129(4):1785−800. doi: 10.1172/JCI96313 30753167 PMC6436884

[13] Erdag G Schaefer JT Smolkin ME Deacon DH Shea SM Dengel LT. Immunotype and immunohistologic characteristics of tumor-infiltrating immune cells are associated with clinical outcome in metastatic melanoma. Cancer Res (2012) 72(5):1070−80. doi: 10.1158/0008-5472.CAN-11-3218 22266112 PMC3306813

[14] Pitt JM Kroemer G Zitvogel L. Immunogenic and non-immunogenic cell death in the tumor microenvironment. Adv Exp Med Biol (2017) 1036:65−79. doi: 10.1007/978-3-319-67577-0_5 29275465

[15] Achard C Surendran A Wedge ME Ungerechts G Bell J Ilkow CS. Lighting a fire in the tumor microenvironment using oncolytic immunotherapy. EBioMedicine (2018) 31:17−24. doi: 10.1016/j.ebiom.2018.04.020 29724655 PMC6013846

[16] Chaput N De Botton S Obeid M Apetoh L Ghiringhelli F Panaretakis T. Molecular determinants of immunogenic cell death: surface exposure of calreticulin makes the difference. J Mol Med Berl Ger (2007) 85(10):1069−76. doi: 10.1007/s00109-007-0214-1 17891368

[17] Niavarani S Lawson C Boudaud M Simard C Tai L. Oncolytic vesicular stomatitis virus-based cellular vaccine improves triple-negative breast cancer outcome by enhancing natural killer and CD8 + T-cell functionality. J Immunother Cancer (2020) 8(1). doi: 10.1136/jitc-2019-000465 PMC7073779 32179632

[18] Lemos de Matos A Franco LS McFadden G. Oncolytic viruses and the immune system: The dynamic duo. Mol Ther Methods Clin Dev (2020) 17:349−58. doi: 10.1016/j.omtm.2020.01.001 32071927 PMC7015832

[19] Garg AD Galluzzi L Apetoh L Baert T Birge RB Bravo-San Pedro JM. Molecular and translational classifications of DAMPs in immunogenic cell death. Front Immunol (2015) 6:588. doi: 10.3389/fimmu.2015.00588 26635802 PMC4653610

[20] Trujillo JA Sweis RF Bao R Luke JJ. T Cell-inflamed versus non-T cell-inflamed tumors: A conceptual framework for cancer immunotherapy drug development and combination therapy selection. Cancer Immunol Res (2018) 6(9):990−1000. doi: 10.1158/2326-6066.CIR-18-0277 30181337 PMC6145135

[21] Prestwich RJ Errington F Steele LP Ilett EJ Morgan RSM Harrington KJ. Reciprocal human dendritic cell-natural killer cell interactions induce antitumor activity following tumor cell infection by oncolytic reovirus. J Immunol Baltim Md 1950 (2009) 183(7):4312−21. doi: 10.4049/jimmunol.0901074 19734207

[22] Marra A Viale G Curigliano G. Recent advances in triple negative breast cancer: the immunotherapy era. BMC Med (2019) 17(1):90. doi: 10.1186/s12916-019-1326-5 31068190 PMC6507064

[23] Keenan BP Jaffee EM. Whole cell vaccines–past progress and future strategies. Semin Oncol (2012) 39(3):276−86. doi: 10.1053/j.seminoncol.2012.02.007 22595050 PMC3356993

[24] Alkayyal AA Tai LH Kennedy MA de Souza CT Zhang J Lefebvre C. NK-cell recruitment is necessary for eradication of peritoneal carcinomatosis with an IL12-expressing maraba virus cellular vaccine. Cancer Immunol Res (2017) 5(3):211−21. doi: 10.1158/2326-6066.CIR-16-0162 28159747

[25] Lemay CG Rintoul JL Kus A Paterson JM Garcia V Falls TJ. Harnessing oncolytic virus-mediated antitumor immunity in an infected cell vaccine. Mol Ther J Am Soc Gene Ther (2012) 20(9):1791−9. doi: 10.1038/mt.2012.128 PMC3437573 22760544

[26] Hanna MG. Immunotherapy with autologous tumor cell vaccines for treatment of occult disease in early stage colon cancer. Hum Vaccines Immunother (2012) 8(8):1156−60. doi: 10.4161/hv.20740 PMC3551893 22854664

[27] Schirrmacher V. Clinical trials of antitumor vaccination with an autologous tumor cell vaccine modified by virus infection: Improvement of patient survival based on improved antitumor immune memory. Cancer Immunol Immunother CII (2005) 54(6):587−98. doi: 10.1007/s00262-004-0602-0 15838708 PMC11042470

[28] Oh J Barve M Matthews CM Koon EC Heffernan TP Fine B. Phase II study of vigil® DNA engineered immunotherapy as maintenance in advanced stage ovarian cancer. Gynecol Oncol (2016) 143(3):504−10. doi: 10.1016/j.ygyno.2016.09.018 27678295

[29] Tai LH de Souza CT Bélanger S Ly L Alkayyal AA Zhang J. Preventing postoperative metastatic disease by inhibiting surgery-induced dysfunction in natural killer cells. Cancer Res (2013) 73(1):97−107. doi: 10.1158/0008-5472.CAN-12-1993 23090117

[30] Tai LH Tanese de Souza C Sahi S Zhang J Alkayyal AA Ananth AA. A mouse tumor model of surgical stress to explore the mechanisms of postoperative immunosuppression and evaluate novel perioperative immunotherapies. J Vis Exp JoVE (2014) 85). doi: 10.3791/51253 PMC4146339 24686980

[31] Hellemans J Vandesompele J. qPCR data analysis – unlocking the secret to successful results. PCR Troubleshooting and Optimization: The Essential Guide Caister Academic Press 2011, ISBN 978-1-904455-72-1

[32] Vandesompele J De Preter K Pattyn F Poppe B Van Roy N De Paepe A. Accurate normalization of real-time quantitative RT-PCR data by geometric averaging of multiple internal control genes. Genome Biol (2002) 3(7):RESEARCH0034. doi: 10.1016/j.ygyno.2016.09.018 12184808 PMC126239

[33] Schmid P Rugo HS Adams S Schneeweiss A Barrios CH Iwata H. Atezolizumab plus nab-paclitaxel as first-line treatment for unresectable, locally advanced or metastatic triple-negative breast cancer (IMpassion130): Updated efficacy results from a randomised, double-blind, placebo-controlled, phase 3 trial. Lancet Oncol (2020) 21(1):44−59. doi: 10.1016/S1470-2045(19)30689-8 31786121

[34] Nanda R Chow LQM Dees EC Berger R Gupta S Geva R. Pembrolizumab in patients with advanced triple-negative breast cancer: Phase ib KEYNOTE-012 study. J Clin Oncol Off J Am Soc Clin Oncol (2016) 34(21):2460−7. doi: 10.1200/JCO.2015.64.8931 PMC6816000 27138582

[35] Slingluff CL Petroni GR Yamshchikov GV Barnd DL Eastham S Galavotti H. Clinical and immunologic results of a randomized phase II trial of vaccination using four melanoma peptides either administered in granulocyte-macrophage colony-stimulating factor in adjuvant or pulsed on dendritic cells. J Clin Oncol Off J Am Soc Clin Oncol (2003) 21(21):4016−26. doi: 10.1200/JCO.2003.10.005 14581425

[36] Senzer N Barve M Kuhn J Melnyk A Beitsch P Lazar M. Phase I trial of « bi-shRNAi(furin)/GMCSF DNA/autologous tumor cell » vaccine (FANG) in advanced cancer. Mol Ther J Am Soc Gene Ther (2012) 20(3):679−86. doi: 10.1038/mt.2011.269 PMC3293620 22186789

[37] Nemunaitis J Barve M Orr D Kuhn J Magee M Lamont J. Summary of bi-shRNA/GM-CSF augmented autologous tumor cell immunotherapy (FANG^TM^) in advanced cancer of the liver. Oncology (2014) 87(1):21−9. doi: 10.1159/000360993 24968881

[38] Guillerme JB Boisgerault N Roulois D Ménager J Combredet C Tangy F. Measles virus vaccine-infected tumor cells induce tumor antigen cross-presentation by human plasmacytoid dendritic cells. Clin Cancer Res Off J Am Assoc Cancer Res (2013) 19(5):1147−58. doi: 10.1158/1078-0432.CCR-12-2733 23339127

[39] Bianchini G De Angelis C Licata L Gianni L. Treatment landscape of triple-negative breast cancer - expanded options, evolving needs. Nat Rev Clin Oncol (2022) 19(2):91−113. doi: 10.1038/s41571-021-00565-2 34754128

[40] Bakos O Lawson C Rouleau S Tai LH. Combining surgery and immunotherapy: Turning an immunosuppressive effect into a therapeutic opportunity. J Immunother Cancer (2018) 6(1):86. doi: 10.1186/s40425-018-0398-7 30176921 PMC6122574

[41] Newman JH Chesson CB Herzog NL Bommareddy PK Aspromonte SM Pepe R. Intratumoral injection of the seasonal flu shot converts immunologically cold tumors to hot and serves as an immunotherapy for cancer. Proc Natl Acad Sci USA (2020) 117(2):1119−28. doi: 10.1073/pnas.1904022116 31888983 PMC6969546

[42] Rosato PC Wijeyesinghe S Stolley JM Nelson CE Davis RL Manlove LS. Virus-specific memory T cells populate tumors and can be repurposed for tumor immunotherapy. Nat Commun (2019) 10(1):567. doi: 10.1038/s41467-019-08534-1 30718505 PMC6362136

[43] Bourgeois-Daigneault MC Roy DG Aitken AS El Sayes N Martin NT Varette O. Neoadjuvant oncolytic virotherapy before surgery sensitizes triple-negative breast cancer to immune checkpoint therapy. Sci Transl Med (2018) 10(422):eaao1641. doi: 10.1126/scitranslmed.aao1641 29298865

