# Supplementary material for: Heterologous prime-boost cellular vaccination induces potent antitumor immunity against triple negative breast cancer
Source: Front Immunol. 2023 Feb 13;14:1098344. doi: 10.3389/fimmu.2023.1098344 (PMC9968850; doi:10.3389/fimmu.2023.1098344)
Supplement: Supplementary file 1 [file Presentation_1.pptx]

## Slide 1
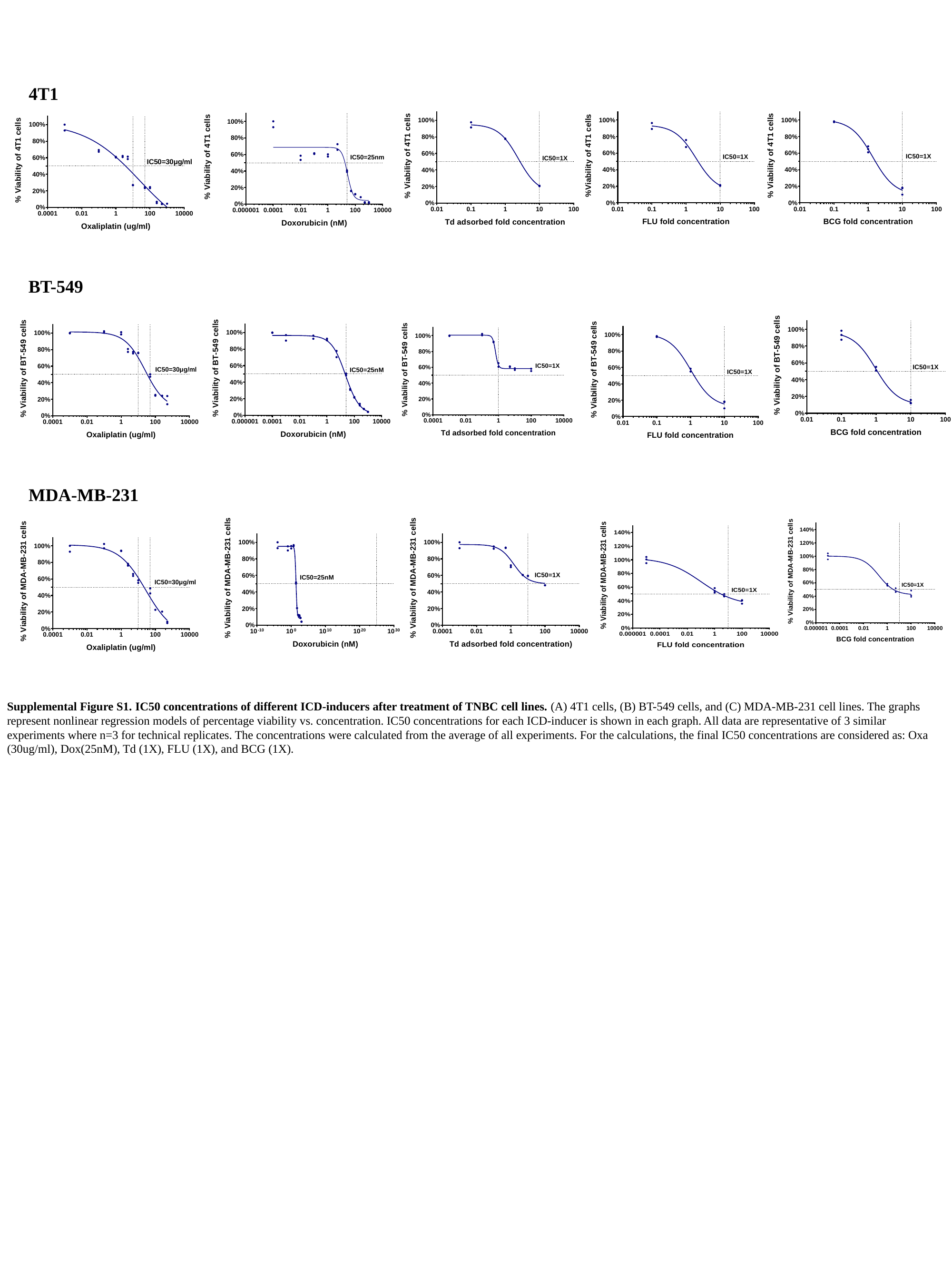

4T1
BT-549
MDA-MB-231
Supplemental Figure S1. IC50 concentrations of different ICD-inducers after treatment of TNBC cell lines. (A) 4T1 cells, (B) BT-549 cells, and (C) MDA-MB-231 cell lines. The graphs represent nonlinear regression models of percentage viability vs. concentration. IC50 concentrations for each ICD-inducer is shown in each graph. All data are representative of 3 similar experiments where n=3 for technical replicates. The concentrations were calculated from the average of all experiments. For the calculations, the final IC50 concentrations are considered as: Oxa (30ug/ml), Dox(25nM), Td (1X), FLU (1X), and BCG (1X).

## Slide 2
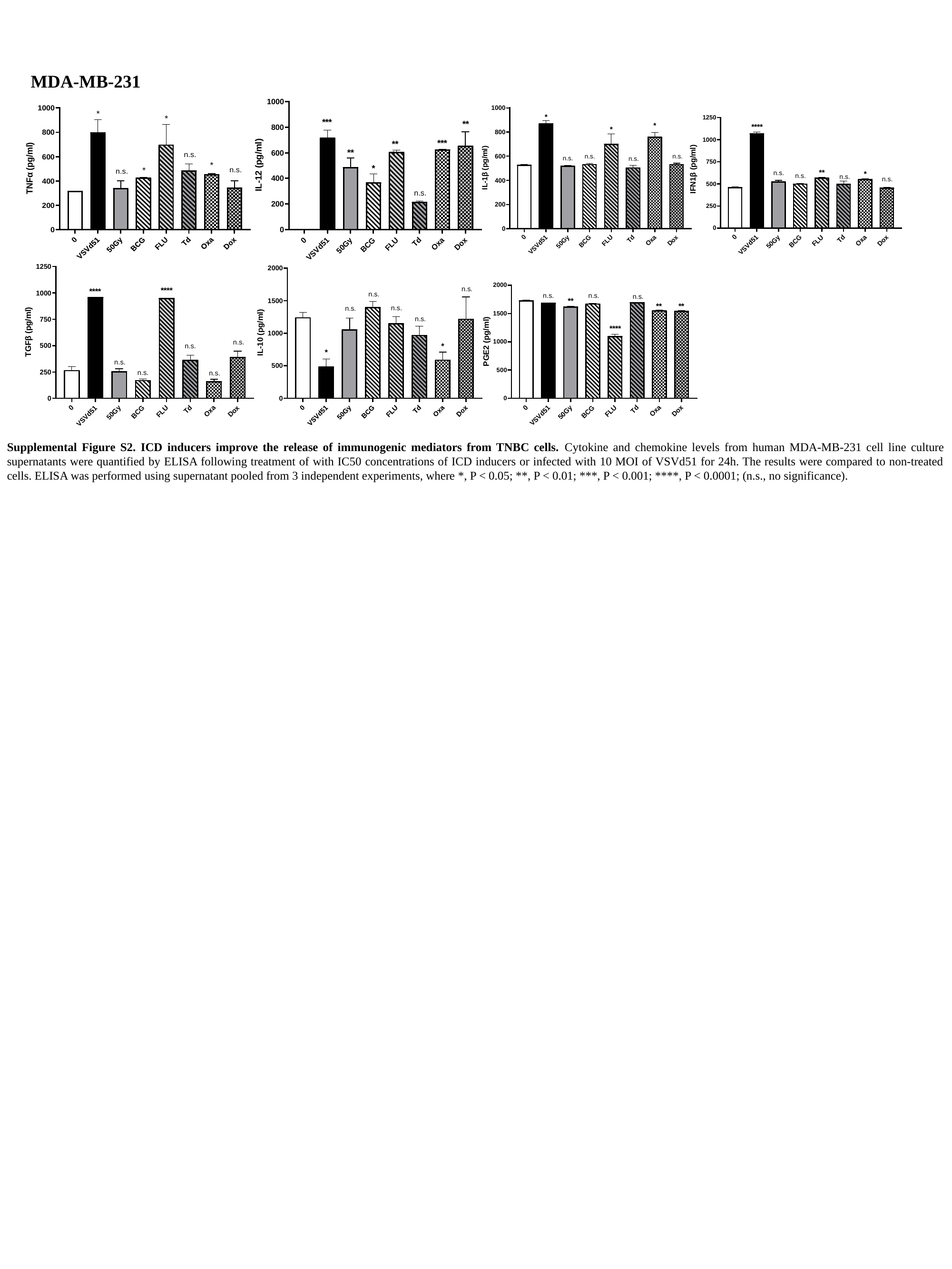

MDA-MB-231
Supplemental Figure S2. ICD inducers improve the release of immunogenic mediators from TNBC cells. Cytokine and chemokine levels from human MDA-MB-231 cell line culture supernatants were quantified by ELISA following treatment of with IC50 concentrations of ICD inducers or infected with 10 MOI of VSVd51 for 24h. The results were compared to non-treated cells. ELISA was performed using supernatant pooled from 3 independent experiments, where *, P < 0.05; **, P < 0.01; ***, P < 0.001; ****, P < 0.0001; (n.s., no significance).

## Slide 3
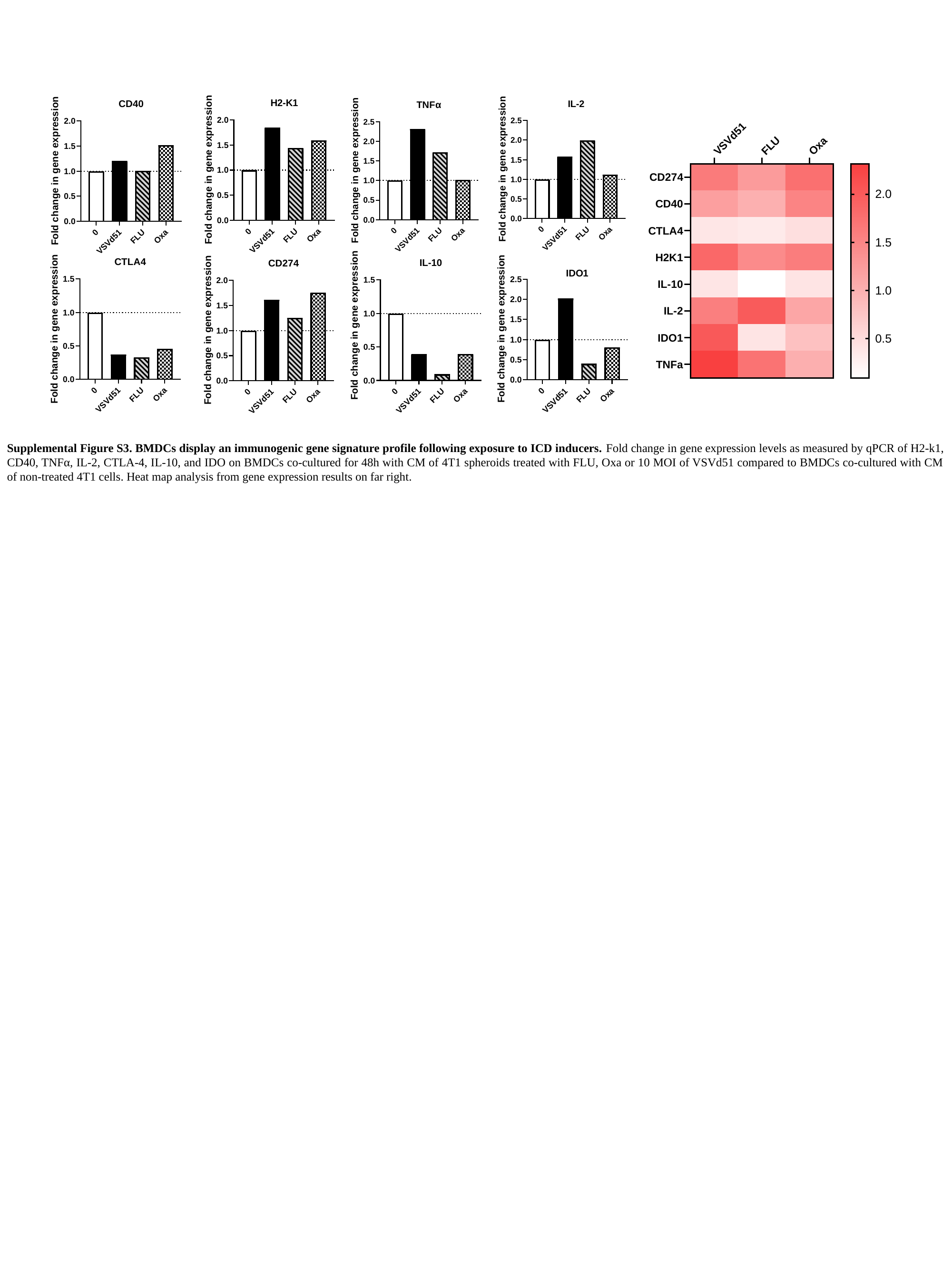

Supplemental Figure S3. BMDCs display an immunogenic gene signature profile following exposure to ICD inducers. Fold change in gene expression levels as measured by qPCR of H2-k1, CD40, TNFα, IL-2, CTLA-4, IL-10, and IDO on BMDCs co-cultured for 48h with CM of 4T1 spheroids treated with FLU, Oxa or 10 MOI of VSVd51 compared to BMDCs co-cultured with CM of non-treated 4T1 cells. Heat map analysis from gene expression results on far right.
